# Cancer Preventive Activities of Tea Catechins

**DOI:** 10.3390/molecules21121679

**Published:** 2016-12-09

**Authors:** Chung S. Yang, Hong Wang

**Affiliations:** Department of Chemical Biology, Ernest Mario School of Pharmacy, Rutgers, The State University of New Jersey, Piscataway, NJ 08854-8020, USA; howang@rci.rutgers.edu

**Keywords:** tea catechins, EGCG, cancer signaling, animal models, cell lines

## Abstract

Catechins are widely occurring in our diet and beverages. The cancer-preventive activities of catechins have been extensively studied. Of these, (−)-epigallocatechin-3-gallate (EGCG), the principal catechin in green tea, has received the most attention. The inhibitory activities of tea catechins against carcinogenesis and cancer cell growth have been demonstrated in a large number of laboratory studies. Many mechanisms for modulating cancer signaling and metabolic pathways have been proposed based on numerous studies in cell lines with EGCG, the most active tea catechin. Nevertheless, it is not known whether many of these mechanisms indeed contribute to the anti-cancer activities in animals and in humans. Human studies have provided some results for the cancer preventive activities of tea catechins; however, the activities are not strong. This article reviews the cancer preventive activities and mechanisms of action of tea catechins involving their redox activities, biochemical properties and binding to key enzymes or signal transduction proteins. These mechanisms lead to suppression of cell proliferation, increased apoptosis and inhibition of angiogenesis. The relevance of the proposed mechanisms for cancer prevention are assessed in the light of the situation in vivo. The potential and possible problems in the application of tea and tea-derived products for cancer prevention are discussed.

## 1. Introduction

In the past 25 years, the cancer-preventive activities of dietary catechins have been studied in a variety of animal models in different laboratories, and catechins from green tea have received the most attention (reviewed in [1,2,3,4,5]). Numerous studies with different cell lines have also been carried out in an attempt to understand the mechanisms of the anti-cancer actions of catechins. This article will use tea catechins as examples to illustrate the extent of research and the challenges ahead of us. Most of the reviewed studies were carried out with (−)-epigallocatechin-3-gallate (EGCG), the most abundant tea catechin. This is a very active area of research. A PubMed search in August 2016 using the keywords “tea and cancer” yielded 4155 citations (1962 to 2016), while “EGCG and cancer” yielded 1435 publications (1989 to 2016). EGCG has been shown to affect a variety of cancer signaling pathways. While it is surprising that a single molecule such as EGCG can have such diverse activities, it is also unclear whether these proposed mechanisms are indeed involved in the inhibition of carcinogenesis or tumor growth in animal models and humans.

This article reviews the biochemical properties of tea catechins, their activities on cancer prevention and possible mechanisms involved. Results from our own laboratory are discussed in more details to serve as examples to illustrate the different approaches used and the challenges in the application of laboratory results for practical application in humans.

## 2. Chemistry, Bioavailability and Biotransformation of Tea Catechins

**Figure 1:**
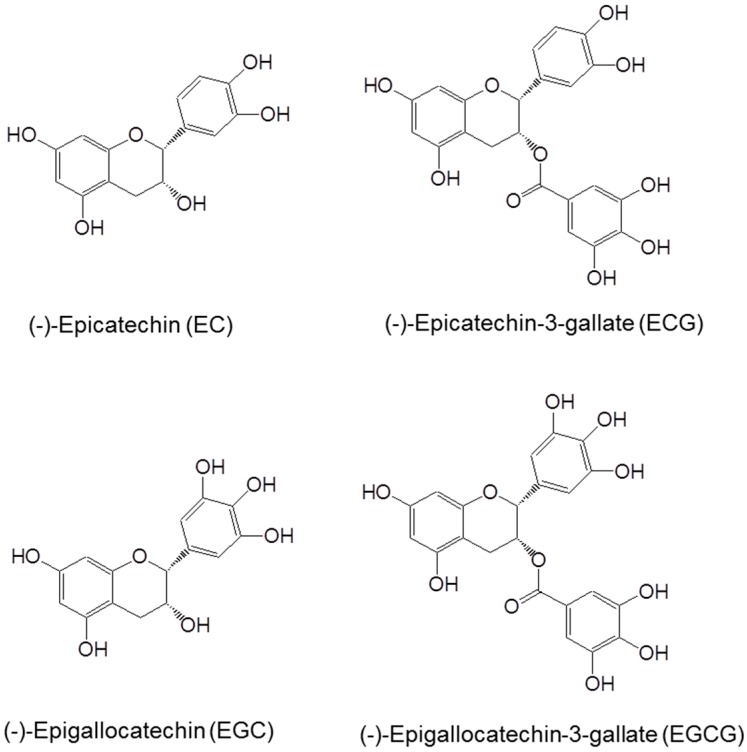
Structures of (−)-epigallocatechin-3-gallate (EGCG), (−)-epicatechin-3-gallate (ECG), (−)-epigallocatechin (EGC) and (−)-epicatechin (EC).

The major catechins in green tea are shown in Figure 1. The polyphenolic structure allows electron delocalization, conferring the ability to quench free radicals. Tea catechins, such as EGCG, have been shown to reduce reactive oxygen species (ROS) such as superoxide radical, singlet oxygen, hydroxyl radical, peroxyl radical, nitric oxide, nitrogen dioxide and peroxynitrite [6]. Among tea catechins, EGCG is most effective in reacting with the majority of ROS. Tea polyphenols are also strong chelators of metal ions such that the chelation of free metal ions prevents the oxidation of catechins and the formation of ROS. The vicinal dihydroxy or trihydroxy structures not only contribute to the antioxidant activity of tea catechins, but also increase their susceptibility to air oxidation under alkaline or neutral pH, especially in the presence of trace amounts of cuprous or ferric ion. Auto-oxidation of EGCG can generate superoxide anion and hydrogen peroxide and leads to the formation of catechin dimers, (such as theasinensins) which are unstable [7,8]. We proposed that this is due to superoxide anion-mediated chain reactions outside of the cells, because EGCG can be stabilized by the addition of superoxide dismutase (SOD) [8]. Such auto-oxidation occurs under cell culture conditions, and the ROS generated by EGCG can induce many cellular changes and cause cell death [1,2,8].

EGCG and other tea catechins undergo extensive biotransformations (reviewed in [9]). Because of the catechol structure, EGCG and other catechins are readily methylated by catechol-*O*-methyltransferase as a detoxification mechanism. In addition, catechins are glucuronidated by UDP-glucuronosyltransferases and sulfated by sulfotransferases. These enzymes, and thus the reactions, occur mainly in the small intestine and liver. Multiple methylation and conjugation reactions can occur on the same molecule [6]. Tea catechins can also be degraded in the intestinal tract by microorganisms. After ingestion of tea catechins by humans, ring fission metabolites5-(3′,4′,5′-trihydroxyphenyl)-γ-valerolactone(M4), 5-(3′,4′-dihydroxyphenyl)-γ-valerolactone (M6) and 5-(3′,5′-dihydroxyphenyl)-γ-valerolactone (M6′) have been observed in urine and plasma samples [10]. These compounds can undergo further degradation to phenylacetic and phenylpropionic acids in the intestine and are excreted in feces and urine. It has been estimated that a large portion of the ingested catechins is degraded by gut microbes [11].

EGCG and other catechins are thought to enter cells through passive diffusion. However, the involvement of transporters, such as organic anion-transporting peptides (OATP) 1A2 and 1B3, has been suggested [12,13]. Active efflux has been shown to limit the bioavailabilities of many polyphenolic compounds, including catechins. The multidrug resistance-associated protein 2 (MRP2), located on the apical surface of the intestine and liver, mediates the transport of some polyphenolic compounds to the lumen and bile, respectively [14]. The involvement of MRP2 in the efflux of EGCG has been reported [15]. EGCG and its metabolites are predominantly effluxed from the enterocytes into the intestinal lumen or from the liver to the bile and excreted in the feces, with little or none of these compounds excreted in the urine [6]. 

The polyphenolic structure of tea polyphenols makes them good donors for hydrogen bonding. Hydrogen bonding of water molecules to EGCG forms a large hydration shell, which reduces the absorption of EGCG. The bioavailability of tea polyphenols fits the Lipinski’s Rule of Five [16] and is dependent on the molecular size, apparent size (due to the formation of a hydration shell) and polarity. For example, the bioavailability of EC (molecular weight 290 and five phenolic groups) is much higher than that of EGCG (molecular weight 458 and eight phenolic groups). In humans, following the oral administration of the equivalent of two cups of decaffeinated green tea, peak plasma levels of EGCG (including the conjugated forms) were usually approximately 0.2 μM [9]. With high pharmacological oral doses of EGCG, peak plasma concentrations of 2–9 μM and 7.5 μM were observed in mice and humans, respectively [17,18].

## 3. Inhibition of Carcinogenesis by Tea or Tea Catechins in Animal Models and Possible Mechanisms

Tea and constituents have been shown to inhibit tumorigenesis in animal models for cancers of the oral cavity, esophagus, stomach, small intestine, colon, liver, pancreas, lung, bladder, prostate, mammary glands and skin [1,2]. Most of the studies were conducted with green tea extracts, tea catechin mixtures or pure EGCG, administered through drinking water or diet. Some examples of these studies are as follows.

### 3.1. Inhibition of Tumorigenesis in the Digestive Tract

The rather low systemic bioavailability of gallated catechins (EGCG and ECG) is a limiting factor for their effectiveness against tumorigenesis in the internal organs. The epithelial cells in the digestive tract have the opportunity of being directly exposed to orally ingested catechins. Inhibitory effects of tea catechins against tumorigenesis in chemical- and genetic-induced animal models in the oral cavity, esophagus, stomach, small intestine, and colon have been shown in more than 30 studies. For example, we showed that administration of EGCG at 0.02%–0.32% in drinking water dose-dependently inhibited small intestinal tumorigenesis in *Apc*^Min/+^ mice, while caffeine did not have an inhibitory effect [19]. The inhibition was associated with increased levels of E-cadherin on the plasma membrane, as well as decreased levels of nuclear levels of β-catenin, c-Myc, phospho-AKT, and phospho-ERK1/2 in the tumors as determined immunohistochemistry (IHC) [19]. Administration of green tea extracts (0.6% in drinking fluid) also inhibited the formation of azoxymethane (AOM)-induced aberrant crypt foci (ACF) in CF-1 mice on a high-fat diet [20]. In another set of experiments, treatment of rats with 0.24% of Polyphenon E (PPE, a standard tea catechin preparation containing 65% EGCG and other tea catechins) in the diet for 8 weeks decreased the total number of ACF in the colon of AOM treated rats. In ACF with high-grade dysplasia, the inhibitory activity of PPE was associated with decreased levels of nuclear β-catenin and cyclin D1, and increased levels of retinoid X receptor-α [21]. In male C57BL/KsJ-*db*/*db* mice, Shimizu et al. [22] demonstrated the inhibition of AOM-induced ACF formation by EGCG (0.01% and 0.1% in drinking water), which was associated with suppression of insulin-like growth factor 1 (IGF1) signaling. The elevated levels of IGF1 receptor (IGF1R), phospho-IGF1R, phospho-GSK3β and β-catenin in the colonic mucosa were decreased by EGCG treatment; also decreased were the plasma levels of IGF1, insulin, triglyceride, cholesterol and leptin [22]. 

There have been suggestions that mixtures of catechins are more effective cancer preventive agents than pure EGCG due to synergistic actions. This hypothesis was tested in *Apc*^Min/+^ mice by comparing the activities of PPE, EGCG, and ECG administered in drinking fluid [23]. The tumor multiplicity was decreased approximately 50% by both PPE (0.12%) and the corresponding amount of dietary EGCG (0.08%), and no difference was observed between PPE and EGCG. On the other hand, the inhibitory activity of ECG was not statistically significant. Examination of the tissues showed that PPE or EGCG treatment increased apoptosis, suppressed cell proliferation, and decreased the levels of phospho-AKT and nuclear β-catenin [23]. Additional studies are required to further determine whether EGCG can interact with other catechins in PPE to generate synergistic actions to inhibit tumorigenesis in other models. In similar studies, we also found that the inhibitory activity of PPE was higher when administered in the diet than in the drinking water, both at 0.12% [23]. 

### 3.2. Inhibition of Lung Tumorigenesis

The inhibitory effects of tea catechins against lung tumorigenesis have been demonstrated in at least 20 studies using chemically-induced and transgenetic rodent models [1,2]. Administration of EGCG or EGC significantly decreased lung tumorigenesis in rats, mice or hamsters [1,2,24]. Oral administration of 0.5% PPE or 0.044% caffeine in the drinking water, to tumor-bearing A/J mice [induced by a single dose of 4-(methylnitrosamino)-1-(3-pyridyl)-1-butanone administered 20 weeks earlier] for 32 weeks, inhibited the progression of lung adenomas to adenocarcinomas [24]. IHC analysis showed that PPE and caffeine treatment inhibited cell proliferation, enhanced apoptosis, and decreased levels of c-Jun and phospho-ERK1/2 in adenocarcinomas. In normal lung tissues, neither agent had a significant effect on cell proliferation or apoptosis, suggesting that the action is selective against tumor tissues. These and other studies demonstrate the broad inhibitory activity of tea catechins against lung carcinogenesis as well as the cancer preventive effects of caffeine. However, the mechanisms of action of these agents remain to be further elucidated.

In other studies, Chung and coworkers demonstrated that caffeine was effective, but not as effective as the corresponding concentration of EGCG as in green tea, in inhibiting lung tumorigenesis in A/J mice [25]. They also showed that the inhibitory effect of caffeine (680 ppm) was similar to that of 2% black tea (containing 680 ppm caffeine) against NNK-induced lung tumorigenesis in rats, suggesting that caffeine was responsible for the inhibitory effect [26]. This conclusion is different from the experiments with A/J mice, which demonstrated the inhibition of lung tumorigenesis by decaffeinated green and black tea preparations [27]. A possible interpretation of this difference is that the systemic bioavailability of tea polyphenols in mice are much higher than in rats, and green tea polyphenols are more bioavailable than the high-molecular-weight polyphenols in black tea [9]. That is, green tea catechins are bioavailable in the mouse lung; whereas much lower levels of black tea polyphenols are bioavailable in the rat lung; therefore, caffeine is likely the major lung cancer preventive agent in black tea extracts. These experiments demonstrate the importance of studies on bioavailability. 

### 3.3. Prostate Carcinogenesis

In transgenic adenocarcinoma of the mouse prostate (TRAMP) mice, administration of a green tea polyphenol infusion (0.1% in drinking water) for 24 weeks markedly inhibited prostate cancer development and distant site metastases [28,29]. The inhibition was associated with decreased cell proliferation, increased apoptosis, decreased IGF1 level, and restored IGF binding protein 3 (IGFBP3) levels in both serum and the dorso-lateral prostate [28,29]. This modulation of IGF1 and IGFBP3 levels was associated with reduced levels of phosphotidylinositol 3-kinase (PI3K) as well as phospho-AKT and phospho-ERK1/2. The green tea polyphenol treatment also significantly decreased levels of angiogenic and metastatic markers, such as vascular endothelial growth factor A (VEGFA), matrix metalloproteinase (MMP)2 and MMP9. These results suggest that the inhibition of the IGF1 signaling, VEGFA and MMPs contributes to the cancer preventive activity of green tea polyphenols. 

## 4. Human Studies on Tea and Cancer

### 4.1. Epidemiological Studies on Tea Consumption and Cancer Risk

Many epidemiological studies have been conducted concerning the cancer-preventive effect of tea consumption in humans, but the results have not been consistent. This is different from the strong evidence for the cancer-preventive activity of tea constituents in animal models [1,2]. In designing animal studies, the experimental conditions, including the doses, are selected to maximize the opportunity to prove the hypothesis. In epidemiological studies, lifestyle factors, genetic differences and other interfering factors reduce the power to detect a cancer-preventive effect. For example, the protective effect of green tea consumption against upper-gastrointestinal cancer became clear after adjusting for interfering factors [30]. Smoking appears to be a strong interfering factor. In a case-control study on the effect of green tea consumption on esophageal cancer in Shanghai by Gao et al. [31], a protective effect was only observed in non-smokers, who were mostly women. A recent systematic review of cohort studies in Japan on green tea consumption and gastric cancer showed no overall preventive effect of green tea. However, a small consistent risk reduction was found in nonsmoking women, and the result became statistically significant after pooling data of six cohort studies [32]. Nevertheless, the above results suggest the cancer preventive effect of tea in humans is only mild. 

### 4.2. Human Intervention Studies with Tea

Intervention trials are important in demonstrating a beneficial health effect of an agent. The results of human intervention studies with green tea polyphenols, mostly small randomized clinical trial (RCT), however, have been inconsistent. For example, an earlier RCT on oral cancer prevention in China, with a mixed tea product (3 g/day administered orally or topically) in patients with oral mucosa leukoplakia for 6 months, showed significant decrease in the number and total volume of proliferation index and silver-stained nucleoli organizer regions [33]. However, a later phase II RCT in the U.S. with green tea extract (500, 750 or 1000 mg/m^2^, 2 times daily) for 12 weeks, to patients with oral pre-malignant lesions (*n* = 28), only showed possible beneficial effects nonsignificant in lessening oral pre-malignant lesions [34]. In an RCT in Japan, subjects with a history of colorectal adenoma, supplementation with green tea extracts (1.5 g/day) for one year significantly decreased the recurrence of adenomas [35]. In this study, the patients were regular tea drinkers (average of 6 cups/day); the dose-response relationship of this study is rather puzzling.

The most impressive intervention study with tea catechins was conducted in Italy, in which 30 men with high-grade prostate intraepithelial neoplasia (PIN) were given 300 mg of green tea catechins, twice daily for 12 months [36]. Only one subject developed prostate cancer, whereas nine of the 30 subjects with high-grade PIN in the placebo group developed prostate cancer. The difference is highly statistically significant. However, a recent trial in Florida with a similar design using PPE (containing 400 mg of EGCG) in 97 men with high-grade PIN and/or atypical small acinar proliferation (ASAP), supplementation for 6 to 12 months did not cause a reduction in the number of prostate cancer cases between the treatment and the placebo groups [37]. Some recent intervention studies on breast cancer and esophageal adenocarcinoma were mainly on the bioavailability and some biomarker [38,39]. At present, the earlier optimistic expectation of cancer preventive activity by tea polyphenols, based on laboratory results, has not materialized in RCTs.

As for therapeutic applications, only a few clinical trials demonstrated the usefulness of EGCG. For example, in a phase 2 trial in patients with early chronic lymphocytic leukemia, oral doses of PPE (2000 mg twice daily) caused durable declines in the absolute lymphocyte count and/or lymphadenopathy in the majority of patients [40]. Side effects observed in this study include transaminitis, abdominal pain and diarrhea. 

## 5. Possible Mechanisms of Cancer Prevention by Tea Catechins

**Figure 2:**
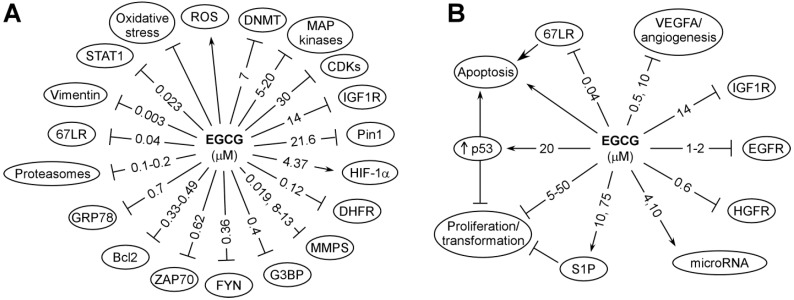
Possible targets for the cancer preventive activity of EGCG (**A**) and subsequent cellular events (**B**). Some of these are direct binding targets; others are affected indirectly. The reported effective concentrations, in IC_50_, K_i_ (inhibition constant) or K_d_ (dissociation constant) are shown in μM. All these are from studies in vitro. When two values are given, the first value is from cell-free systems and the second value is from studies in cell lines (modified from [3]).

The primary actions of catechins are due to their redox and physical binding activities, and this topic has been reviewed [3,5]. Using EGCG as an example, these actions are depicted in Figure 2 and discussed below. 

### 5.1. Antioxidant and Pro-Oxidative Activities and Carcinogen Metabolism

Catechins are well recognized as antioxidants, but they can also be pro-oxidants and generate ROS. ROS can alter the functions of cellular proteins, lipid and nucleic acids, and lead to different diseases [41]. Oxidative damages to DNA cause mutation and genomic instability, which are major contributing factors in the initiation, promotion and progression of carcinogenesis [42]. Although the antioxidant activity of tea catechins is well established in vitro [6], such activity in vivo is only observed under circumstances when the animals are under oxidative stress; for example, in old rats (but not in young rats) [43] and in smokers [44]. In animal models for carcinogenesis, ROS are induced by the treatment with carcinogens, and EGCG has been demonstrated to reduce the formation of 8-hydroxydeoxyguanosine (8-oxo-dG), a well-established marker for oxidative DNA damage that can mispair to induce mutations [25]. As endogenously formed ROS are important in promoting carcinogenesis, tea catechins may have important roles in quenching these species at different stages of carcinogenesis. In human studies, administration of green tea to smokers for 4 weeks has been shown to significantly reduce the number of 8-oxo-dG-positive cells [44]. Such antioxidant actions of tea catechins may decrease the risk of carcinogenesis.

Tea catechins can be auto-oxidized to generate ROS in cell culture medium and cause cell death [8,45]. After entering the cells, high concentrations of EGCG may also induce the production of ROS by a different mechanism, possibly involving the electron transport chain in the mitochondria [46]. In our studies, oral administration of EGCG to mice bearing human lung cancer H1299 cell xenograft tumors inhibited tumor growth, enhanced tumor cell apoptosis, and produced ROS in the tumor cells [47]. The observed ROS accumulation in tumor cells is probably due to the lack of sufficient antioxidant enzymes in H1299 cells. It remains to be demonstrated whether the production of ROS is responsible for the induction of apoptosis in vivo. At modest doses (e.g., 0.5% EGCG in the diet), although increased levels of 8-oxo-dG and γ-H2AX (phosphorylated histone 2A variant X) were seen in xenograft tumors, 8-oxo-dG production and toxicity were not observed in the liver, kidney and other organs of the host mice [47].

Cellular ROS may also activate the nuclear factor erythroid 2-related factor 2 (Nrf2)-mediated signaling pathways to induce cytoprotective enzymes [48]. For example, oral gavage of EGCG (200 mg per kg) to C57BL/6J mice upregulated gene expression of γ-glutamyltransferase, glutamate cysteine ligase and haemoxygenase 1 in the liver and colon, which were most likely mediated by the activation of Nrf2 [49]. Similarly, human volunteers supplemented with 800 mg PPE per day for 4 weeks increased glutathione *S*-transferase P activity in lymphocytes [50]. In an intervention study in a high aflatoxin exposure area in China, supplementation with 500 or 1000 mg green tea polyphenols per day for 3 months increased the median urinary aflatoxin B1-mercapturic acid levels by more than 10-fold compared to baseline [51]. This result is likely due to the induction of glutathione *S*-transferase by EGCG. It appears that levels of ROS produced by moderate doses of tea polyphenols activate Nrf2 to reduce oxidative stress. However at high doses (e.g., 750 mg/kg, ig.), EGCG can produce high levels of ROS and induce hepatotoxicity [52]. These toxic responses are probably similar to the reported liver toxicity in individuals who took excessive amounts of tea extracts in dietary supplements used for the purpose of weight reduction [53,54]. 

### 5.2. High Affinity Binding to Protein Targets

EGCG is known to bind to a variety of proteins with rather high affinities, through multiple hydrogen bonding to active site proteins. In our previous work with molecular modeling, the binding of EGCG to DNA methyltransferase (DNMT) 1 has been proposed to involve five hydrogen bonds [55]. In an earlier study, using NMR spectroscopy, EGCG was demonstrated to directly bind to the BH3 pocket of anti-apoptotic Bcl2 proteins—with inhibition constant (K_i_) of 0.33–0.49 μM [56]. However, higher EGCG concentrations (by two orders of magnitude) were needed to induce apoptosis. 

Using an EGCG-Sepharose 4B column and 2D-gel electrophoresis, Dong et al. identified vimentin [57], IGF1R [58], FYN [59], glucose-regulated protein 78 kDa (GRP78) [60], ZAP70 [61] and Ras-GTPase-activating protein SH3 domain-binding protein 1 (G3BP1) [62] as high-affinity EGCG binding proteins (Figure 2). A subsequent X-ray crystallography study demonstrated the binding of EGCG to both the WW and PPIase domains of peptidyl prolyl *cis*/*trans* isomerase (Pin1) [63]. The direct binding of EGCG with Pin1 was confirmed and the binding inhibited Pin1 PPIase activity. This inhibition could have important biological consequences because Pin1 is required for full activation of AP-1, NFκB, β-catenin and other signaling pathways. Biochemical studies showed a dissociation constant of 21.6 μM for the binding of EGCG to Pin1. EGCG was shown to suppress the proliferation of cells expressing Pin1 and tumor growth in a xenograft mouse model Pin1 [63]. 

Studies with surface plasmon resonance (SPR), molecular modeling and site directed mutagenesis found that EGCG (and ECG) could bind tightly to signal transduction activator of transcription 1 (STAT1) with K_d_ of 23 nM in MDA-MB-231 breast cancer cells [64]. The binding involved at least three hydroxyl groups of the B ring and one hydroxyl group of the D ring of EGCG. Site-directed mutagenesis of STAT1 with H568A eliminated the high-affinity binding to EGCG. The tight binding of EGCG to STAT1 blocked its phosphorylation by Janus kinase 2 (JAK2). This could be a mechanism by which EGCG inhibits STAT1 activation and related actions on cytokines and growth factors. EGCG was also suggested to inhibit the JAK/STAT3 signaling and lead to Fas/CD95-mediated apoptosis of head and neck squamous carcinoma cells [65]. 

All of the aforementioned proteins were demonstrated to be important for the inhibitory activity of EGCG in some cell lines. However, much higher EGCG concentrations (than the K_d_ values) were needed to elicit the expected cellular response. For example, vimentin bound to EGCG with a K_d_ of 3.3 nM, and studies in cultured cells showed that EGCG inhibited the phosphorylation of vimentin with IC_50_ = 17 μM. It is possible that EGCG binds nonspecifically to proteins and other macromolecules in the cells and therefore elevate the effective concentrations of EGCG at the targets. The discovery of the aforementioned high-affinity EGCG-binding proteins is important, and the direct involvement of these proteins in the action of EGCG in animal models and humans remains to be investigated.

### 5.3. Inhibition of Enzyme Activities

Tea polyphenols have been shown to bind and inhibit the activities of a variety of enzymes. In many studies, the inhibition of enzyme activity has been observed in cultured cells and the inhibition could be due to either direct binding or indirect actions. We previously observed that EGCG, at concentrations of 5–20 μM, inhibited the phosphorylation of JNK (Jun N-terminal kinase), c-Jun, MEK1/2, ERK1/2 and ELK1 in JB6 epidermal cell lines [66]. This inhibition was associated with the inhibition of AP-1 transcriptional activity or cell transformation. Additional studies with in vitro kinase assays suggested that EGCG inhibited MEK1/2 phosphorylation by decreasing its association with the kinase RAF1 [67]. Moreover, EGCG seemed to inhibit the phosphorylation of ELK1 by competing with the binding site for ERK1/2 [68]. Recent studies also suggest that EGCG inhibited the phosphorylation of ERK1/2 and AKT in Epstein-Barr virus (EBV)-positive cells, and this could block the constitutive lytic infection of EBV at the gene transcription and translation levels [69]. There were also studies showing that EGCG activated ERK1/2 and other MAP kinases through the generation of ROS, but these results could be in vitro artifacts. In lung carcinogenesis models, EGCG and a green tea polyphenol preparation have been shown to inhibit the phosphorylation of c-Jun and ERK1/2 [24]. 

EGCG has also been reported to inhibit the chymotryptic activity of 20S proteasomes [70], which is a key step in the degradation of many signaling proteins. The difference in the effective concentrations of EGCG in cell-free systems (IC_50_ = 0.09–0.2 μM) and in cell lines (IC_50_ = 1–10 μM) [70], is probably due to nonspecific binding to different macromolecules. EGCG and other catechins have been shown to inhibit the activity of secreted MMP2 and MMP9 with IC_50_ values of 8–13 μM [71,72]. MMPs are secreted by tumor cells during cancer cell invasion and metastasis. Recent studies also demonstrated that EGCG induced TIMP3 by epigenetic mechanisms in MCF7 and MDA-MB 231 breast cancer cells [73]. It remains to be demonstrated whether these activities contribute to inhibit metastasis and invasion in vivo.

We reported previously that EGCG inhibited DNMT1 activity (K_i_ ~ 7 μM) in cancer cell lines, and this resulted in the demethylation and reactivation of the hypermethylated promoters of the tumor suppressor gene INK4A, retinoic acid receptor β, as well as the DNA repair genes, MLH1 and methylguanine methyltransferase [55]. Reactivation of some of these genes was also observed in HT29 colon and PC3 prostate cancer cells. Tea catechins also reactivated GSTP1 in human prostate cancer cells by causing promoter hypomethylation and chromatin remodeling [74]. However, the induction of promoter demethyaltion by EGCG was not observed in some other hypermethylated genes. As will be discussed in a later section, EGCG has been suggested to induce other epigenetic events. EGCG has also been reported to inhibit dihydrofolate reductase [75], glucose-6-phosphate dehydrogenase [76] glyceraldehyde-3-phosphate dehydrogenase [77], and carbonyl reductase 1 [78]. These results on enzyme inhibition are interesting. Their relevance in vivo, especially concerning specificity to target enzymes in cancer cells versus normal cells, remain to be elucidated.

### 5.4. Effects on 67kDa Laminin Receptor (67LR)

Binding of EGCG to the 67LR (with a K_d_ value of 0.04 μM) was first observed by Tachibana et al. using a SPR assay [79]. Expression of the metastases-associated 67LR increased the responsiveness of MCF7 cells to low micromolar concentrations of EGCG [79]. RNA interference (RNAi)-mediated silencing of 67LR abrogated EGCG-induced apoptosis in multiple myeloma (MM) cells [80]. Further studies in cultured macrophages showed that 67LR mediated anti-inflammation action of EGCG [81]. Anti-67LR antibody treatment or RNAi-mediated silencing of 67LR resulted in the abrogation of the inhibitory action of EGCG on lipopolysaccharide-induced activation of TLR4 and downstream signaling of inflammation. Recent work by Kumazoe et al. [82] showed that the activation of 67LR by EGCG in primary MM cells and MM cell lines resulted in elevated levels of cGMP to initiate apoptosis. However, EGCG alone was not very effective in killing MM U266 cells (IC_50_ of 23.2 μM), because these cells overexpressed phosphodiesterase 5 (PDE5), which degrades cGMP. When a PDE5-selective inhibitor, vardenafil, was also added to cultured cells, it synergized with EGCG to reduce the IC_50_ of EGCG to 1.4 μM. This impressive synergism was also shown in a xenograft model, as well as in vitro in some breast, gastric, pancreatic and prostate cancer cell lines, which overexpresses both 67LR and PDE5 [82].

### 5.5. Inhibition of Receptor Tyrosine Kinases and Other Receptors

Tea catechins have been shown to affect many receptor-mediated activities. Their inhibitory actions against receptor tyrosine kinases (RTKs) have been reviewed recently by Larsen et al. [83] and Shimizu et al. [84]. All members of the RTK family, including epidermal growth factor (EGFR), IGF1R, hepatocyte growth factor receptor (HGFR or c-Met) and vascular endothelial growth factor receptor (VEGFR), consist of an extracellular ligand-binding domain, single membranes-spanning region and a cytoplasmic protein tyrosine kinase domain. The major signaling pathways activated by RTKs are the Ras/ERK and the PI3K/AKT pathways. Members of the EGFR family are frequently overexpressed in cancers and are associated with poor prognosis [85]. Many studies have demonstrated the inhibitory effects of EGCG on the EGFR signaling pathways [8,86,87,88,89]. Several mechanisms have been proposed for the inhibition of EGFR by EGCG: (a) interfering with the binding of EGF to EGFR and inhibiting EGFR tyrosine kinase activity [86]; (b) altering lipid organization in the plasma membrane (lipid rafts) and inhibiting EGF binding to EGFR [88]; and (c) inducing EGFR internalization without activation [89]. The synergistic action of EGCG and erlotinib, an EGFR tyrosine kinase inhibitor, against head and neck cancer cell growth has been reported [90]. Inhibition of EGFR signaling has also been shown to decrease the production of VEGFA in cancer cells [91]. In addition, EGCG (0.5–10 μM) has been shown to disrupt VEGFA-induced VEGFR2 dimerization in human umbilical vein endothelial cells [92], and inhibited growth and activation of VEGF/VEGFR axis in human colorectal cancer cells [93]. In a murine gastric tumor model, EGCG suppressed VEGFA protein expression and tumor microvessel density [94].

IGF1R activation by IGF1 can induce cell proliferation, cell survival, transformation, metastasis and angiogenesis as well as inhibit apoptosis in different cancer cell lines [95]. IGF/IGF1R axis has been reported to be targets of EGCG in human colon and hepatocellular carcinoma cells [96,97]. EGCG also inhibits IGF1R phosphorylation in human colon cancer SW837 cells [96]. Direct binding of EGCG to IGF1R [58], as discussed previously, is a likely mechanism. These results from cell line studies are consistent with those from animal studies showing that orally administrated EGCG and other tea catechins inhibited the IGF/IGF1R axis in a colon carcinogenesis model in *db*/*db* obese mice [22] and TRAMP mice [28]. 

Deregulation of HGFR pathway occurs in several types of human cancers and can lead to increased tumorigenesis and metastasis [98]. HGF and HGFR play key roles in epithelial–mesenchymal transition, which is associated with tumor invasion [99]. It has been shown in MDA-MB-231 cells that the HGF-induced phosphorylation of HGFR, and AKT is completely blocked by 0.6 μM EGCG, and that cell invasion is significantly decreased by 5 μM EGCG [100]. Larsen et al. has provided evidence for the binding of EGCG to the ATP-binding site of HGFR [101]. In a series of non-small cell lung cancer cell lines, EGCG was also found to be a potent inhibitor of cell proliferation and appeared to be more effective against HGFR than against EGFR [102]. EGCG has also been suggested to transcriptionally target sphingosine-1-phosphate receptor S1P2 and prevent sphingosine-1-phosphate mediated signaling in HL-60 promyclomoncytic leukemia cells [103].

### 5.6. Inhibition of Wnt Signaling

The Wnt signaling involves the nuclear translocation of β-catenin to transcriptionally activate proto-oncogenes such as c-Myc, cyclin D1 and COX-2. Our studies in *Apc*^min/+^ mice suggest that EGCG inhibits Wnt signaling [13]. Treatment of HT29 human colon cancer cells with EGCG (20 μM) decreased nuclear levels of β-catenin as well as cellular levels of c-Myc and cyclin D1 [13]. Our recent studies in colon cancer cell lines also suggested that EGCG induced β-catenin *N*-terminal phosphorylation at the Ser33/37 residues and promoted its degradation in *Apc* mutated colon cancer cell lines [104]. The EGCG-induced β-catenin phosphorylation and degradation is consistent with a similar observation by Singh et al. [105]. EGCG was also shown to inhibit the Wnt signaling in hepatoblastoma cells [106]. Interestingly, this was found to be associated with the reexpression of the silenced tumor suppressor gene, secreted frizzled-related protein (SFRP)1, which is known to modulate Wnt signaling

### 5.7. Other Mechanisms

#### 5.7.1. Epigenetic Mechanisms 

In addition to the aforementioned epigenetic changes [55,73,74,106], EGCG was reported to decrease the levels of 5-methylcytosine, DNMT activity, and expression levels of DNMT1, DNMT3a and DNMT3b in human epidermoid carcinoma A431 cells. It also decreased HDAC activity and affected levels of acetylated lysines on histones H3 and H4 [107]. Furthermore, EGCG inhibited acetyltransferase (HAT) enzymes [108] and inhibited the transcription of hTERT (human telomerase reverse transcriptase), the catalytic subunit of telomerase, through epigenetic mechanisms mediated at least partially through the inhibition of DNMT and HAT activities [109]. 

#### 5.7.2. Effect on microRNA

MicroRNAs are small (about 22 bases) single-stranded, endogenous noncoding RNAs that negatively regulate the translation and/or stability of mRNAs [110]. MicroRNA levels could be altered by EGCG to cause subtle changes in multiple molecular targets and pathways. It has been reported that EGCG upregulated miR-16 in HepG2 cells, and this led to the downregulation of Bcl2 and induction of apoptosis [111]. In our recent work in both human and mouse lung cancer cells in culture, we found that EGCG specifically upregulated the expression of mir-210, a major microRNA regulated by HIF-1α [112]. The upregulation of mir-210 was found to be correlated with the transiently stabilized HIF-1α in lung cancer cell lines after EGCG treatment. We also demonstrated that EGCG could bind to the oxygen-dependent degradation (ODD) domain of the hypoxia-response element of HIF-1α promoter and prevented the hydroxylation-dependent ubiquitination and proteasome-mediated degradation of HIF-1α. The in vivo relevance of this observation, however, remains to be demonstrated. A recent study also showed that EGCG upregulated miR-16 in breast cancer cell line 4T1 [113]. The miR-16 could be transfected to tumor-associated macrophages (TAM) via exosomes and inhibited TAM infiltration and M2 microphage polarization. These actions were suggested to be responsible for the observed growth suppression of xenograft tumors from 4T1 cells in BALB/c mice treated with EGCG.

#### 5.7.3. Modulation of p53-Dependent Events

Many studies have demonstrated that EGCG treatment induces p53 expression and p53-dependent apoptosis: however, different mechanisms have been suggested in different cell line studies [114,115,116,117]. The involvement of p53 in the biological activity of EGCG requires additional studies in vitro and in vivo.

#### 5.7.4. Binding to Lipids

The possibility that EGCG alters lipid organization in the plasma membrane (lipid rafts) and affect protein distribution and receptor functions has been proposed for the inhibition of the functions of EGFR [88], HGFR [118], and 67LR [119]. Although interesting, it remains to be determined whether the effects occur in normal cells, whether EGCG also alters the lipid rafts of cancer cells in vivo, and what concentrations of EGCG are required to exert an observable effect in vivo. 

#### 5.7.5. Binding to Nucleic Acids

Based on the physical binding of EGCG to nucleic acids, it has been suggested that DNA and RNA can also be targets of action of tea catechins [120]. However, the relevance of this proposed binding depends on whether the catechins can bind selectively to specific nucleic acid in the genome of cancer or premalignant cells without affecting normal cells.

### 5.8. Issues in Extrapolating Studies In Vitro to Situations In Vivo

Most of the information discuss above was derived from studies in cell lines, mainly with EGCG. It is unclear why a molecule such as EGCG can influence so many signaling pathways and whether these activities are manifested in vivo. Some studies have also included other catechins; their activities generally follow the ranking order of EGCG > ECG > EGC > EC. 

In relating observations in vitro to molecular events in vivo, an important issue is the different concentrations used. In most animal cancer prevention studies, the EGCG levels in the blood and tissues were usually lower than 0.5 µM. How do we evaluate the relevance of an experiment using 10–100 μM EGCG in cell cultural studies? To address the relationship between the effective concentrations in vivo versus in vitro, we compared the EGCG levels in blood and xenograft tumors of H1299 lung cancer cells. At conditions when tumor growth was inhibited approximately 50% by EGCG (0.5% in the diet), the EGCG concentrations was 0.52 μM in blood and 0.18 μM in tumor tissues. This value was 2-orders of magnitude lower than the IC_50_ values of EGCG in the inhibition of H1299 cell growth in culture [47]. One possible reason for the observed discrepancy between the cell culture system and the xenograft model is the rather short-term exposure to EGCG in cell culture studies (24 or 48 h) compared to the long-term treatment in animal models. Prolonging the treatment period of cells in culture has been shown to reduce the effective concentration of EGCG [87]. The environment for cells in culture is also very different from that in tumors. Therefore, we cannot rule out a mechanism just because the in vitro effective concentrations of EGCG are higher than we observed in vivo. However, it is reasonable to assume that activities effected by low concentrations of EGCG are likely to be more relevant than activities that are produced only at higher concentrations. The results from very high concentrations of catechins in cell culture systems may not be relevant to cancer prevention. Similarly, events due to ROS generated by EGCG extracellularly may not occur in vivo [8]. To avoid such a problem, we suggest the inclusion of SOD and catalase in the incubation mixture to prevent or minimize extracellular ROS generation. 

## 6. Concluding Remarks

The above discussions on the cancer-preventive activity of tea are based mostly on studies with green tea and green tea polyphenols. This will serve as a basis for us to understand the cancer- preventive activities of other teas. For example, black teas are expected to have lower cancer- preventive activities because the black tea polyphenols, theaflavins and thearubigins, have little or no bioavailability. These black tea polyphenols are formed by the polyphenol oxidase-catalyzed oxidation and polymerization of catechins, in a process generally known as fermentation, during the manufacturing of black tea. This prediction is consistent with results from laboratory studies and epidemiological observations [25,26,27,30]. Oolong tea is manufactured by fermentation for a short period of time to allow the formation of oligomers of catechins [6]. Therefore, the cancer preventive activity of oolong tea should be similar to green tea. However, this area has not been extensively studied.

A frequently asked question is whether all the reported actions of catechins are relevant for cancer prevention or cancer therapy in vivo. Apparently, mechanisms suggested by cell line experiments and observed in cancer prevention studies in animal models are likely to be more relevant. These include the induction of apoptosis in different animal models, inhibition of the phosphorylation of c-Jun and ERK1/2 in lung tumorigenesis models, suppression of phospho-AKT and nuclear β-catenin levels in colon cancer models, inhibition of the IGF/IGF1R axis in colon and prostate cancer models, and suppression of VEGF-dependent angiogenesis in lung and prostate cancer models [19,22,24,29,121]. It is still unclear whether these molecules are direct targets for EGCG or downstream events of the primary actions. It is reasonable to assume that some of the high affinity binding proteins as discussed in Section 5 could serve as initial targets, but this point remains to be investigated in animal models.

A rationale for the use of cancer cell lines in cancer prevention research is that mechanisms observed in cancer cells may be applicable to inhibition of carcinogenesis, in particular cancer progression*.* However, the in vivo relevance of some of the reported mechanisms remain to be substantiated. There are some examples of practical application of tea catechins. The use of green tea polyphenols (PPE) ointment (trademarked as VEREGEN^®^) for the treatment of genital warts (*Condyloma acuminatum*) has been approved by the FDA [122]. The key feature for success of this medication is the topical application of catechins which have anti-viral activity. As discussed earlier, clinical trials with PPE (2000 mg EGCG, twice daily for up to 6 months) have shown beneficial effects in patients with chronic lymphocytic leukemia [40]. There were side effects, which may be tolerable in short-term therapies for cancer patients, but are not acceptable for long-term use. Some researchers propose the use of a combination approach, e.g., the use of EGCG in combination with cisplatin or alkylating agents [123]. The EGCG is proposed to enhance drug efficacy and decrease toxicity. The combination of EGCG with cetuximab (antibody against EGFR) has recently been shown to exert strong anti-tumor activity against triple negative breast cancer orthoxenograft, without signs of toxicity [124]. Oral administration of EGCG has been shown to attenuate acute radiation-induced esophagitis in a phase II trial with 37 stage III lung cancer patients [125]. A recent study has also shown that EGCG ameliorated cisplatin-induced renal injury in a mouse model [126]. For reducing drug toxicity by EGCG, the rationale is to reduce the oxidative stress generated by the drug. However, caution should be applied considering EGCG can be a pre-oxidant under certain conditions. The practical application of the combination approaches remains to be demonstrated.

Because of the broad cancer preventive activities of tea catechins in different animal models, multiple mechanisms are likely to be involved. Even in the same experimental system, one tea catechin such as EGCG may exhibit cancer inhibitory activities via more than one mechanism. It is interesting to consider the possibility that these actions may work synergistically to exert the cancer preventive effects. Precise information about the mechanisms of cancer prevention by tea in humans is even more difficult to obtain. From the limited data that are available, actions of tea polyphenols in reducing oxidative stress and enhancing the metabolic elimination of carcinogens [50,51] may be important. Such actions will be difficult to verify in human intervention studies using cancer as an endpoint. Well-designed large cohort studies may shed light on the cancer preventive activity of tea and other catechins. The relationship between tea consumption and cancer risk may become clearer if we better quantify catechin consumption, correct for life-style factors, and consider genetic polymorphisms of the individuals.
